# Fully Automated Synthesis of Novel TSPO PET Imaging Ligand [^18^F]Fluoroethyltemazepam

**DOI:** 10.3390/molecules26082372

**Published:** 2021-04-19

**Authors:** Dario Fiorenza, Emanuele Nicolai, Carlo Cavaliere, Ferdinando Fiorino, Giovanna Esposito, Marco Salvatore

**Affiliations:** 1 IRCCS SDN, 80143 Napoli, Italy; emanuele.nicolai@synlab.it (E.N.); carlo.cavaliere@synlab.it (C.C.); direzionescientifica.irccssdn@synlab.it (M.S.); 2 Department of Pharmacy, School of Medicine and Surgery, University of Naples Federico II, 80131 Naples, Italy; fefiorin@unina.it (F.F.); giovannaesposito10@gmail.com (G.E.)

**Keywords:** translocator protein, benzodiazepine, neuroinflammation, positron emission tomography probe, molecular imaging

## Abstract

Introduction: Benzodiazepines, including temazepam are described as TSPO antagonists. In fact, TSPO was initially described as a peripheral benzodiazepine receptor (PBR) with a secondary binding site for diazepam. TSPO is a potential imaging target of neuroinflammation because there is an amplification of the expression of this receptor. Objectives: Herein, we developed a novel fluorinated benzodiazepine ligand, [^18^F]Fluoroethyltemazepam ([^18^F]F-FETEM), for positron emission tomography (PET) imaging of translocator protein (18 kDa). Methods: [^18^F]F-FETEM was radiolabelled with an automated synthesizer via a one-pot procedure. We conducted a [^18^F]F-aliphatic nucleophilic substitution of a tosylated precursor followed by purification on C18 and Alumina N SPE cartridges. Quality control tests was also carried out. Results: We obtained 2.0–3.0% decay-uncorrected radiochemical activity yield (3.7% decay-corrected) within the whole synthesis time about 33 min. The radiochemical purity of [^18^F]F-FETEM was over 90% by TLC analysis. Conclusions: This automated procedure may be used as basis for future production of [^18^F]F-FETEM for preclinical PET imaging studies.

## 1. Introduction

Context and background: Translocator protein TSPO is a protein located on the outer mitochondria membrane mainly expressed in the brain in glial cells. Mitochondria are involved in several vital and detrimental processes and mitochondrial dysfunctions are related in a variety of neurodegenerative diseases such as Alzheimer’s disease, Parkinson’s disease, Huntington’s disease and psychiatric condition such as anxiety and bipolar disorder [1].

TSPO was initially described as a peripheral benzodiazepine receptor (PBR) with a secondary binding site for diazepam; however, subsequent research has found that this receptor is expressed throughout whole body. In humans, translocator protein is encoded by the TSPO gene and belongs to the tryptophan-rich family of sensory proteins [2].

TSPO-binding ligands showed influence in a variety of cellular functions (i.e., cholesterol transport and steroid hormone synthesis, mitochondrial permeability, apoptosis, proliferation, tumor genesis and inflammation [3,4,5]. In the brain, TSPO increase expression was found in activated microglia during neuroinflammation and immunosuppressive action of the TSPO ligands make TSPO an attractive target for diagnostic imaging [6]. TSPO translocator protein is an 18 kDa protein with five strongly hydrophobic transmembrane domains, found mainly on the outer mitochondrial membrane of microglia [7]. This receptor interacts with StAR (steroidogenic acute regulatory protein) for the transport of cholesterol into mitochondria [3]. In the last few years, numerous PET imaging radiotracers specific for TSPO have been synthesized and reported in the literature labeled with carbon-11 and fluorine-18 [8].

Over the last few years, numerous ligands (and radioligands) for TSPO have been synthesized and reported in the literature [9]:

i. 2-Phenylindolylglyoxylamides;
ii. Quinazoline carboxamides;
iii. Aryloxyanilides;
iv. Benzoxazolone and benzimidazolone derivatives;
v. Imidazopyridine, pyrroloquinoline, pyrazoloquinoline and pyrazolopyrimidine derivatives.

TSPO antagonists, including benzodiazepines temazepam, showed a significant antagonistic properties based on cell proliferation assays. Although benzodiazepines, such as temazepam, are molecules well known in literature and have been used for decades in clinical practice, clinical application as antagonists of TSPO is still in an initial phase (in vitro) [10].

The first radiotracer, used for more than 20 years, was *N*-sec-butyl-1-(2-chlorophenyl)-*N*-[^11^C]-methyl-3-isoquinolinecarboxamide, known by the acronym [^11^C]C-PK11195, pharmacologically a TSPO antagonist, in racemic solution of which the enatiomer (R) is the most active [11].

PK11195 isoquinoline was originally described as a compound that partially displaced some benzodiazepines such as diazepam. In fact, this compound has receptor affinity for peripheral benzodiazepine receptors. Furthermore, PK11195 can be found in mitochondrial fractions of brain extracts and in mitochondria-free erythrocytes.

Clinical use of this tracer for PET imaging is based on three observations: only minimal PK11195 binding is shown in healthy patients; in central nervous system diseases, in neuroinflammatory conditions, binding is found particularly in activated microglia; radiolabelling with carbon-11 allows PET imaging.

Therefore, other radiotracers have also been developed with preclinical and clinical studies, some of which use fluorine-18 as radioisotope which has a longer half-life, compared to carbon-11 (109 min compared to 20 min for carbon-11, according to database of the National Nuclear Data Center (NNDC) at Brookhaven National Laboratory, Upton, NY, USA).

Unknown factors/problems: However, several practical problems arise with TSPO radiotracers: not distinguishing pro- and anti-inflammatory responses, low signal-to-noise ratio, low variation of dynamic response during neurodegenerative diseases, TSPO polymorphisms.

These problems appear to have been reduced and/or eliminated with second and third generation radiotracers; however, there is still no possible differentiation between the M1 (neurotoxic) and M2 (neuroprotective) genotypes of microglia. Furthermore, even if 3D pentameric structure of the TSPO has been revealed, the role of this receptor is not yet clear, as well as the influence of receptor upregulation in immune reaction is not clear. New studies have shown that TSPO radiotracers (especially first and second-generation ones) show three binding affinity patterns derived from the rs6971 polymorphism: high affinity ligands (HABs), mixed affinity ligands (MABs) and low affinity ligands. (LABs) [12]. PET signal provided in MAB and LAB patients leads to a significant underestimation TSPO expression. However, it seems that the third generation TSPO radioligands are not sensitive to the rs6971 polymorphism, such as [^18^F]flutriciclamide ([^18^F]F-GE180); moreover, it would seem that the lesion/background ratio is higher than the other TSPO tracers [13,14].

Experimental approach/major findings: In this paper, we introduce a new type of TSPO radiotracer (3S)-7-chloro-3-[^18^F]fluoroethyl-1-methyl-5-phenyl-3*H*-1,4-benzodiazepin-2-one, [^18^F]Fluoroethyltemazepam ([^18^F]F-FETEM) radiolabelled with an automated synthesizer via a one-pot procedure. Validated methods reported in literature for benzodiazepines radiolabeling (such as [^18^F]Flumazenil) make use of purification methods by semipreparative HPLC. These synthesis methods involve an increase in production times, which can even reach 80 min, with a yield corrected by decay of 15%. Herein, proposed synthesis and purification method achieved a balance between reduction of synthesis times, removal of impurities using SPE cartridges, use of a versatile synthesis module with a non-invasive cleaning procedure and without carryout of products from previous processes.

Implications: [^18^F]F-FETEM can extend our understanding on TSPO implication in neurodegenerative diseases.

## 2. Results

In contrast, to procedure described in literature, we were able to apply a standardfluorination technique that involved preparation of dry K[^18^F]/kryptofix complex following nucleophilic displacement reaction at 120 °C for 5 min in anhydrous DMSO. In order to optimize reaction conditions, temperature, time and solvent were investigated.

An equimolar concentration of [K/K2.2.2]^+^ [^18^F]Fluoride^-^/precursor was used and the incorporation of [^18^F]Fluoride in was found to be 2.0–3.0%. The combination of C18 and alumina cartridges gave the highest yield. C18-concentration step was also applied to obtain the greatest possible percentage of pure product. Crude mixture reaction was transferred to C18 cartridge with purification solution WFI:EtOH 7:3 v/v and DMSO, K222 and unreacted [^18^F]Fluoride do not bind to C18 and are transferred to waste. Final formulation solution was filtered with a Sep-Pak Plus Alumina N directly connected with a sterile filter into the final product vial. Starting from 16.65 ± 1.85 GBq (450 ± 50 mCi) of [^18^F]F^−^, 0.499 ± 0.037 GBq (13.5 ± 1 mCi) of [^18^F]F-FETEM was readily formulated, in a total synthesis time of 33 min in a mean (±standard deviation).

The radiochemical purity was 90% as evaluated by Radio-TLC with Rf 0.80. [^18^F]F-FETEM was stable for 3 h, performing same QC tests for time zero.

## 3. Discussion

The objective of our research was synthesis of the target compound [^18^F]F-FETEM and his corresponding p-toluenesulfonyl precursors (Table 1), required for fluorine-18 labelling. The increased clinical need for TSPO radiotracers prompted us to design an automated device for [^18^F]F-FETEM synthesis.

For the automated synthesis of [^18^F]F-FETEM we used a custom-modified commercially available automated synthesis module for nucleophilic ^18^F-fluorinations TRACERlab™ FXFDG, which is similar to the TRACERlab™ FXN synthesis module, which has been shown to be a versatile synthesis platform for the production of a range of different ^18^F-labelled radiopharmaceuticals [21].

**Figure 1:**
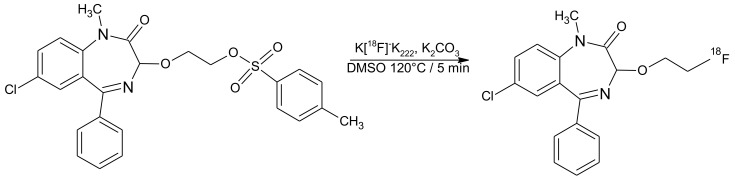
Reaction for [^18^F]F-FETEM using GE Tracerlab FXFDG synthesis module in one step synthesis by classical [^18^F]fluoride nucleophilic substitution of the p-toluenesulfonyl precursor using potassium carbonate and Kryptofix^®^ 222 at 100 °C for 5 min.

**Figure 2:**
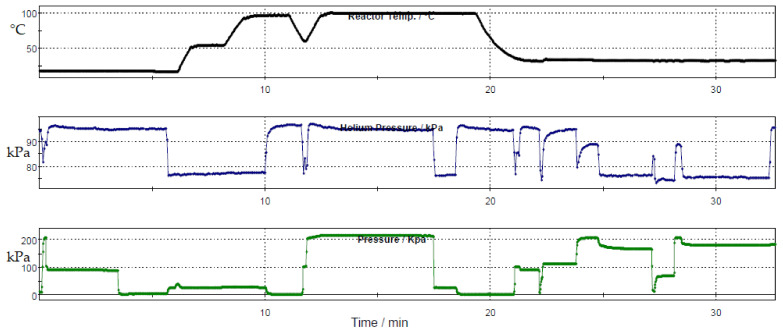
Trendings (temperature, helium pressure, reactor pressure/time) for [^18^F]F-FETEM using GE Tracerlab FXFDG synthesis module.

The radiolabelling of [^18^F]F-FETEM was achieved in one step synthesis by classical [^18^F]fluoride nucleophilic substitution of the p-toluenesulfonyl precursor using potassium carbonate and Kryptofix^®^ 222 at 100 °C for 5 min (Figure 1 and Figure 2) in a closed reactor under helium flow.

**Figure 3:**
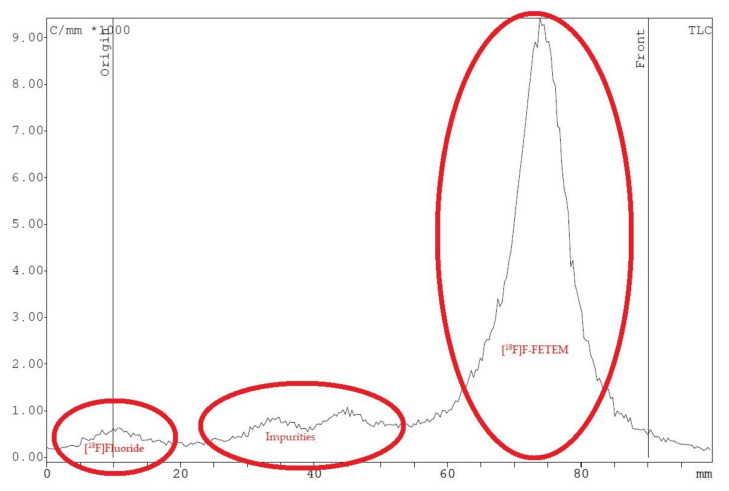
Radio-TLC analysis of radiochemical impurities of [^18^F]F-FETEM.

**Figure 4:**
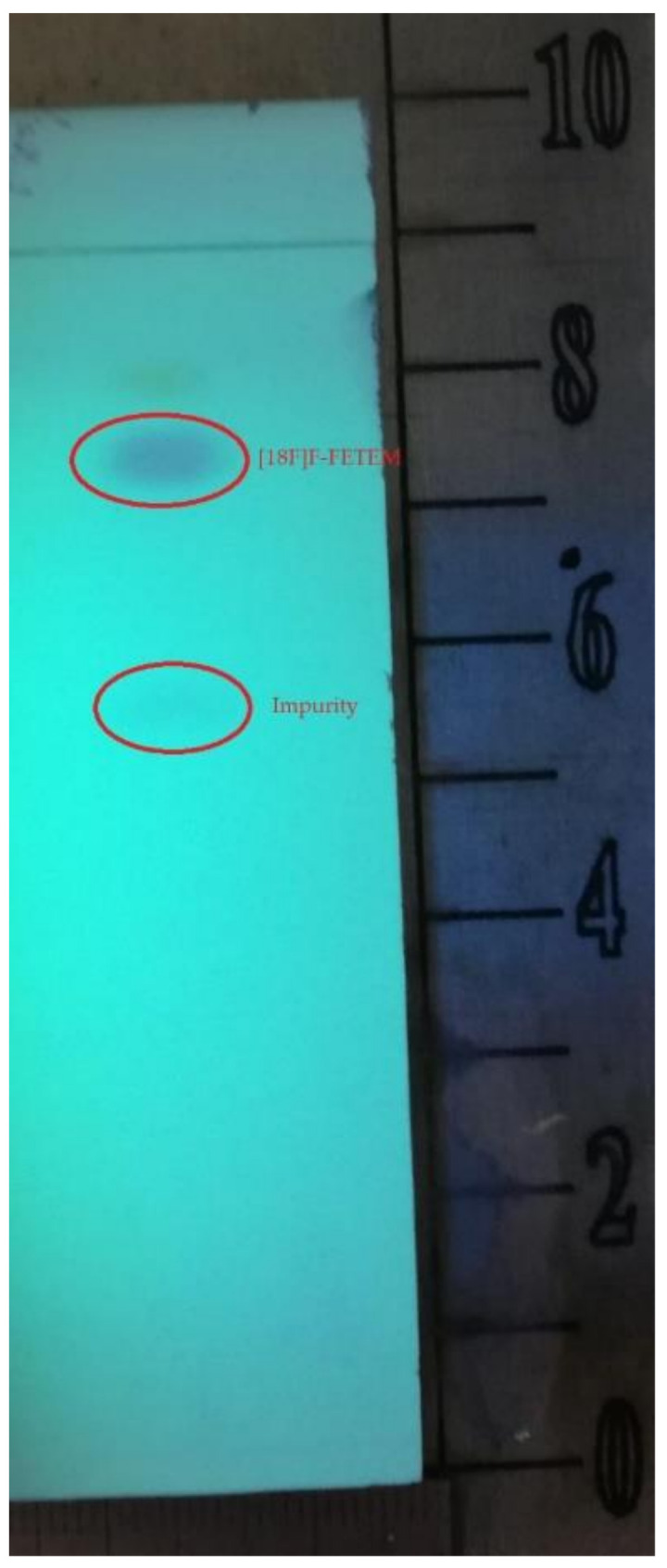
TLC analysis of chemical impurities of [^18^F]F-FETEM detection in UV light at 254 nm.

**Figure 5:**
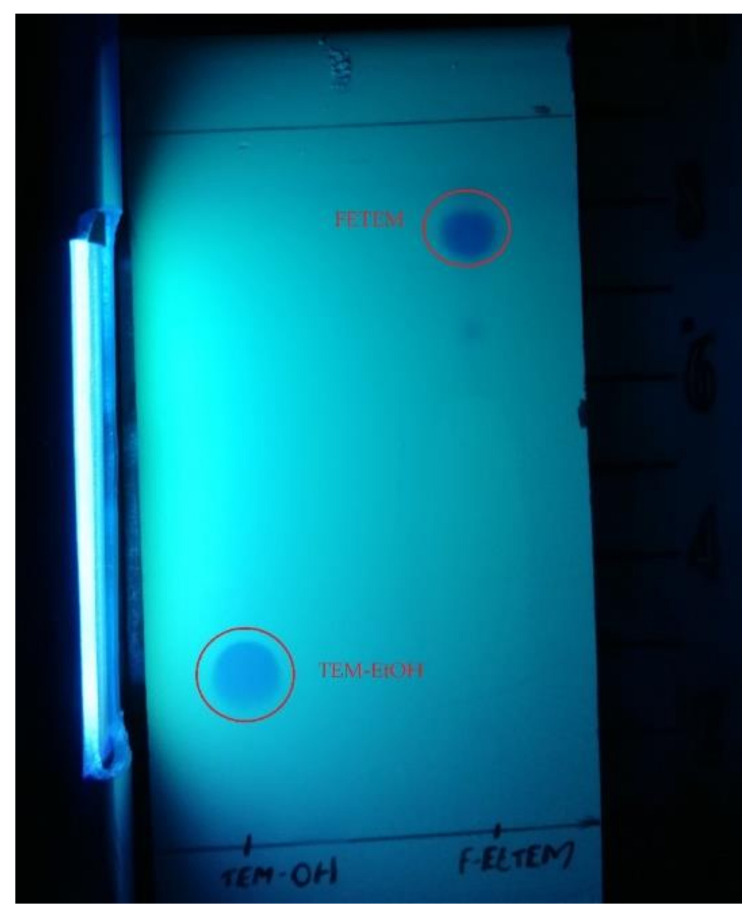
TLC analysis of standards FETEM and TEM-EtOH, detection in UV light at 254 nm.

**Figure 6:**
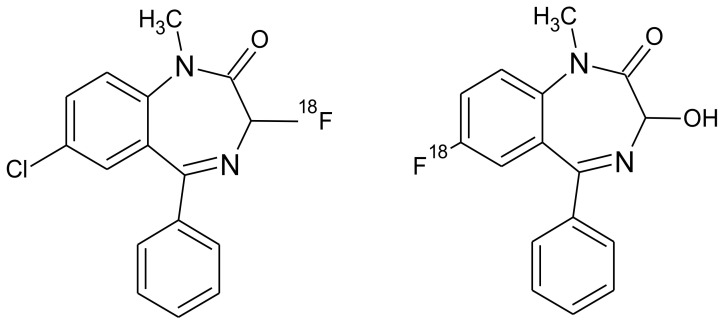
Possible radiochemical impurities of [^18^F]F-FETEM synthesis.

Owing to a high reaction temperature (100 °C) solvent (DMSO) was used with no evaporation during the reaction because of short reaction times (5 min). The Sep-Pak purification provided a simple separation of [^18^F]F-FETEM from radiochemical impurities. After purification and formulation of the radiotracer, results from this study provide evidence an equivalent radiochemical yield (non-decay-corrected) of 2.0–3.0% (3.7% decay-corrected) of [^18^F]F-FETEM. Quality control results show a radiochemical purity of 90% [^18^F]F-FETEM, other 10% is unreacted [^18^F]Fluoride and radioimpurities not yet identified (Figure 3, Figure 4 and Figure 5). Since this TLC chromatographic system has already been verified and reported in literature [22], we can hypothesize that other radiochemical impurities obtained from TLC chromatograms are benzodiazepine molecules shown in Figure 6. 

These impurities derive from radiofluorination directly on benzodiazepine ring, in one case in position 3 (where the leaving group would be OH-EtTos) in another in position 6 (where the leaving group would be a chlorine atom). Radioimpurities do not increase during the stability period verified (3 h). According to preclinical studies data reported for TSPO radiotracers in the literature, this stability of 3 h allows us to perform in vivo biodistribution studies. Furthermore, radioimpurities do not increase in three hours and no decomposition products were found in the three hours analyzed so it allow to perform a longer stability study (6 h). Plus, in vivo preclinical studies are more feasible by using the fluorine-18 radioisotope, compared to carbon-11 (109 min compared to 20 min for carbon-11).

Our study expands on work of Barresi et al. [9] of TSPO ligands as potential therapeutic or diagnostic tools. In fact, in their work, benzodiazepines such as temazepam are not reported as ligands. In the literature, TSPO antagonists, including benzodiazepines temazepam, are reported in vitro but no benzodiazepine ligands have ever been used for PET imaging [10,23]. One potential limitation of future studies is that [^18^F]F-FETEM binds TSPO and GABA receptors at the same time. In addition, we cannot define a yield optimization by varying the amount of precursor, including adjustments of the reaction temperatures and times. In the preclinical production of [^18^F]F-FETEM, we encountered problems with lipophilicity of the [^18^F]F-FETEM. The relatively low radiochemical yield (RCY) is due to radioactivity losses in incomplete radiochemical reactions, as well as to a lesser extent to side reactions. There is room for further optimization. However, we are able to produce sufficient amounts of [^18^F]F-FETEM for preclinical purposes. Further studies are necessary to validate a HPLC method for [^18^F]F-FETEM, we are carrying out validation studies of the HPLC method using the method reported in European Pharmacopoeia Temazepam monograph (01/2008:0954).

In conclusion, our results suggested a new type of benzodiazepine radioligands for TSPO never previously described. However, further studies are necessary to validate [^18^F]F-FETEM in vivo with neurodegenerative diseases animal models.

## 4. Materials and Methods

### 4.1. Chemicals and Materials

1,10-Diaza-4,7,13,16,21,24-hexaoxabicyclo[8,8,8]-hexacosane (Kryptofix 2.2.2, K222), potassium carbonate (K2CO3), acetonitrile (anhydrous, 99.8%) were obtained from ABX GmbH (Radeberg, Germany). Dimethyl sulfoxide (DMSO; anhydrous, ≥99.9%), chloroform (for analysis), acetone (Reag. Ph. Eur.) and ethanol absolute anhydrous (EtOH, Reag. Ph. Eur.) were obtained from Merck Lifescience or Merck Millipore. Sterile water for injection (WFI, Ph. Eur.) was obtained from Galenica Senese (Monteroni d’Arbia, Italy) and helium from SOL (Marcianise, Italy). All reagents and solvents were used as received from the commercial suppliers.

The enriched [18O]H2O (≥98%) for [^18^F]fluoride production was purchased from Rotem Industries Ltd. (Mishor Yamin D.N. Arava, City, Israel).

Solid-phase extraction (SPE) cartridges, Sep-Pak C18, Sep-Pak Plus Alumina N and Accell Plus QMA Carbonate were obtained from Waters Corp. (Milford, MA, USA). Sterile Vented Millex GS syringe filters with mixed cellulose ester membrane (0.22 μm, 27 mm) were obtained from Merck Millipore Ltd. (Cork, Ireland). 

### 4.2. Precursor and Standard Synthesis

Temazepam (EP Reference Standard), *N*,*N*-Dimethylformamide (DMF, anhydrous, 99.8%), thionyl chloride (SOCl2, ReagentPlus^®^, ≥99%), ethylene glycol (ReagentPlus^®^, ≥99%), chloroform (for analysis), p-Toluenesulfonyl chloride (TsCl, ReagentPlus^®^, ≥99%), 4-(Dimethylamino)pyridine (DMAP, ReagentPlus^®^, ≥99%), Triethylamine (TEA, ≥99.5%), silica gel 60 (extra pure for column chromatography), ethyl acetate (anhydrous ≥99.8%) were obtained from Merck Lifescience or Merck Millipore. 

Temazepam (2 g, 0.00665 mol), solubilized in DMF (10 mL), was added to thionyl chloride (3.95 g, 0.0332 mol) in a stoichiometric excess of 5 equiv. Reaction mixture was then kept under electromagnetic stirring at 0 °C whole night. Subsequently, reaction mixture was evaporated under reduced pressure to remove solvent and excess reactive and the product thus obtained (2.393 g, 0.0074 mol) was used without further purification in the subsequent reaction step. 

Ethylene glycol (20 mL) was previously heated to a temperature of 50 °C to which the previously obtained chlorinated intermediate was added. The reaction mixture thus obtained was kept at 50 °C for 15 min. and was cooled to room temperature and it was left for further 4 h under electromagnetic stirring. Subsequently, ice was added to the reaction mixture and an extraction was carried out using chloroform. The organic phase thus obtained was back-extracted with water, anhydrified with anhydrous Na_2_SO_4_, filtered and evaporated. The intermediate thus obtained (2.775 g, 0.00805 mol) was used without further purification in the subsequent reaction step in which it was solubilized in anhydrous DCM (20 mL). To this solution were added tosyl chloride (1.726 g, 0.00905 mol, 1.12 equiv.), DMAP (0.393 g, 0.00321 mol, 0.4 equiv.), TEA (0.814 g, 1.12 mL, 0.00804 mol, 1 equiv.). Resulting mixture was kept under electromagnetic stirring at room temperature overnight. Reaction mixture was evaporated and residue was purified on a silica gel chromatographic column using ethyl acetate/hexane 7:3 v/v as eluent mixture. Finally, the final product thus obtained was recrystallized from hexane and identified by ^1^H-NMR and ^13^C-NMR nuclear magnetic resonance spectroscopy and ESI-MS mass spectrometry.

Addition of ethylene glycol to temazepam (TEM-EtOH) and Tosylation (TEM-EtTos) were performed according to Table 2.

Standard (TEM-EtOH): Yield 95%; mp: 220–221 °C. 1H-NMR (400 MHz, CDCl3) 3.47 (s, 3H, -CH3); 3.87 (m, 2H, -OCH2-); 3.92 (s, 1H, -OH); 4.05 (m, 2H, CH2-OH); 4.85 (s, 1H, -CH-); 7.34 (bs, 1H); 7.49 (m, 3H); 7.53 (d, 1H); 7.58 (dd, 1H); 7.65 (d, 2H). ESI-MS: 345.15 [M+H]+; 367.18 [M + Na]^+^; 483.16 [M + K]^+^ (Calcd: 344.79).

Precursor (TEM-EtTos): Yield 53%; mp: 81–83 °C. 1H-NMR (400 MHz, CDCl3) 2.43 (s, 3H, -CH3); 3.14 (s, 3H, -CH3); 3.66 (m, 2H, -OCH2-); 4.10 (m, 2H, -CH2-O); 5.67 (s, 1H, -CH); 7.49–7.87 (m, 12H). ESI-MS: 499.3 [M + H]^+^; 521.20 [M + Na]^+^; 537.14 [M + K]^+^ (Calcd: 498.98).

### 4.3. Synthesis and Quality Control

[^18^F]Fluoride production was performed in a cyclotron MINItrace GE 9.6MeV. The automated radiosynthesis of [^18^F]F-FETEM was performed in a modified commercial radiosynthesis module TRACERlab FXFDG synthesizer (GE Healthcare, Pollards Wood, Nightingales Lane, Chalfont St Giles, Buckinghamshire, England HP8 4SP). Radiochemical purity was performed with a Raytest miniGITA TLC Scanner (Raytest, Straubenhardt, Germania).

### 4.4. Radiochemistry

Absolute radioactivity measurements were performed using a calibrated ionization chamber (Capintec CRC-25PET).

### 4.5. Preproduction Procedures

Before every synthesis, the synthesizer was cleaned and the lines dried following internal synthesizer-specific written instructions and all disposable items were changed to new ones. K222 (15.0 ± 0.5 mg, 39.84 µmol) was dissolved in CH3CN (1.0 ± 0.1 mL). Precursor (15.0 ± 0.1 mg, 30.06 µmol) was weighted into a 2.5 mL glass vial and dissolved in DMSO (1.0 ± 0.1 mL). Eluent solution EtOH:WFI 7:3 *v*/*v* (4.0 ± 0.1 mL) was measured into a disposable syringe. Next, reagents were loaded into appropriate synthesizer positions as follows: (1) K2CO3 solution (6 mg/mL, 0.55 mL); (2) Kryptofix^®^ 222 (15 mg/1 mL acetonitrile); (3) TEM-EtTos precursor (15 mg/1 mL DMSO); (4) empty; (5) Purification with WFI:EtOH 7:3 *v*/*v* (4.0 ± 0.1 mL); and (6) Eluent solution EtOH:WFI 7:3 *v*/*v* (4.0 ± 0.1 mL). SPE cartridges (Accell Plus QMA, C18 and Plus Long Alumina N) were preconditioned with ethanol (2.0 mL) and sterile water (2.0 mL). Sep-Pak cartridges for purification of the reaction mix was C18. The end-product vial set comprised a sterile pyrogen-free glass vial, Plus Long Alumina N, two sterile filters (Vented Millex^®^-GS syringe filters for end-product filtration and for ventilation) and a syringe for QC sampling. This end-product vial set was labelled and assembled in an isolator (clean room grade A).

### 4.6. [18.F]Fluoride Production

Oxygen-18 enriched water in a silver target was irradiated with 9.6 MeV protons produced with a MINItrace cyclotron. The beam current was 40 μA for 30 min. After the irradiation, the target water with [^18^F]Fluoride was transferred in a target vial of synthesizer via a helium flow at 60 psi.

### 4.7. [18.F]F-FETEM Production

The automated synthesis was controlled and monitored from PC and comprised the following automated steps (Table 3):

- After irradiation, [^18^F]Fluoride was trapped in a preconditioned QMA cartridge.
- Aqueous [^18^F]Fluoride was eluted with 0.55 mL of 6 mg/mL potassium carbonate solution and transferred to the reactor.
- Once aqueous [^18^F]fluoride was transferred to the reactor, Kryptofix^®^ 222 was added to the reactor.
- The dry K222/[^18^F]^−^/K^+^-complex was formed by azeotropic distillation under reduced pressure and helium flow. Reactor was heated for 1.5 min at 97 °C. Controlled vacuum was applied and He flow supplied.
- After azeotropic distillation step, heater and reactor were cooled at 60 °C under He and air flows and controlled vacuum.
- Pre-loaded TEM-EtTos precursor solution (15 mg/1 mL DMSO) was added to reactor. The reactor was heated for 5 min at 100 °C under magnetic stirring. After reaction, reactor was cooled at 33 °C under He and air flows and controlled vacuum.
- After cooling, purification solution WFI:EtOH 7:3 *v*/*v* 4.0 mL was added to reactor.
- Reaction mix was transferred by reactor needle and loaded to C18 Sep-Pak cartridge. Eluate produced by this transfer was sent to the waste bottle.
- After loading the reaction mix onto the purification cartridges, eluent solution EtOH:WFI 7:3 *v*/*v* 4.0 mL was added to reactor and directly sent to purification cartridge. Eluate containing radiopharmaceutical [^18^F]F-FETEM was collected inside the collect vial of the synthesizer.
- [^18^F]F-FETEM was sent to the isolator (grade A), purified on Sep-Pak Plus Long Alumina N cartridge in a sterile pyrogen-free glass vial previously labelled.
- After addition of the formulation solution, integrity test was performed to sterile filter.
- In isolator a QC sample (1 mL) was collected into another sterile pyrogen-free glass vial previously labelled.
- After obtaining the QC sample, all filters and needles were removed from the end-product vial and the radioactivity was measured.

### 4.8. Specifications and Quality Control (QC) Tests

QC tests on the QC sample were performed following the specifications and test methods below:

- Appearance: A clear and yellow solution, free of particles (Method: visual inspection).
- Radiochemical purity (RCP): the fluorine-18 radioactivity in the form [^18^F]F-FETEM is ≥90.0% (Method: TLC).
- Radionuclidic identity: half-life of 105–115 min (Method: measured in dose calibrator).
- Radionuclidic purity: ≥99.9% of the radioactivity corresponds to fluorine-18 (Method: gamma-ray spectrometry).
- pH: 4.0–7.5 (Method: pH indicator strip).

## 5. Conclusions

Here, we described the development of an automated synthesis one-step one-pot reaction for preclinical production of [^18^F]F-FETEM in a single-reactor TRACERlab™ FXFDG synthesis module. Moreover, the entire process is straightforward and this user-friendly device and process provides adequate quantities of [^18^F]F-FETEM with low RCY for preclinical studies.

## Figures and Tables

**Table 1:** Summary of TSPO PET tracers most commonly described in literature.

PET Tracer	Chemical Structure	TSPO Generation	Reference
[^11^C]C-PK11195 (isoquinoline carboxamide ligand)	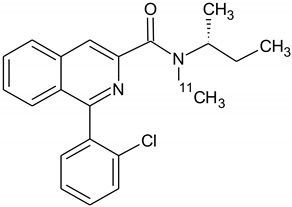	First generation	[15]
[^18^F]F-GE-180 (tetrahydrocarbazole ligand)	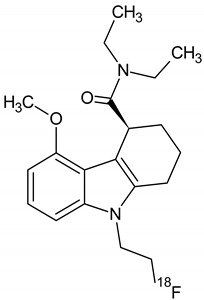	Third generation	[16]
[^18^F]F-DPA-714 (pyrazolepyrimidines ligand)	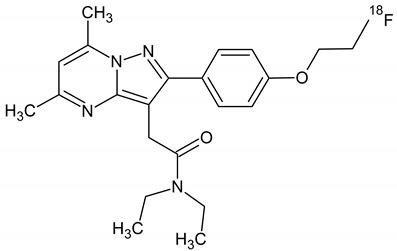	Second generation	[17]
[^18^F]F-PBR06 (phenoxyarylacetamides ligand)	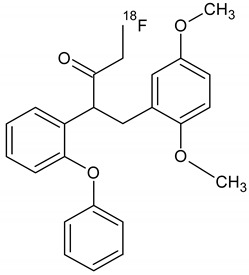	Second generation	[18]
[^11^C]C-PBR28 (phenoxyarylacetamides ligand)	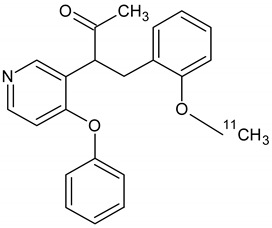	Second generation	[19]
[^18^F]F-FEPPA (phenoxyarylacetamides ligand)	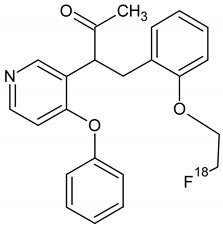	Second generation	[20]

**Table 2:** Summary of the reaction sequence for FETEM precursor synthesis.

Synthesis of (3S)-7-chloro-3-ethylhydroxy-1-methyl-5-phenyl-3*H*-1,4-benzodiazepin-2-one (TEM-EtOH)
1. Addition of thionyl chloride (5 equiv., 3.95 g, 0.0332 mol) to temazepam (2 g, 0.00665 mol) in DMF (10 mL) under magnetic stirring over night (0 °C) 2. Evaporation of solvent 3. Addition of ethylene glycol (20 mL at 50 °C) to the reactor vial 4. Reaction at 50 °C for 15 min and 4 h at room temperature under magnetic stirring 5. Extraction in chloroform 6. Evaporation of organic phase
**Synthesis of (3S)-7-chloro-3-ethyltosyl-1-methyl-5-phenyl-3*H*-1,4-benzodiazepin-2-one (TEM-EtTos)**
7. Addition of tosyl chloride (1.12 equiv., 1.726 g, 0.00905 mol), TEA (0.814 g, 1.12 mL, 0.00804 mol, 1 equiv.) and DMAP (0.393 g, 0.00321 mol, 0.4 equiv.) to TEM-EtOH (2.775 g, 0.00805 mol) in DCM (20 mL) under magnetic stirring over night (room temperature) 8. Evaporation of solvent 9. Purification of TEM-EtTos with silica gel column 10. TEM-EtTos desorption by the eluent (ethyl acetate:hexane 7:3) 11. Recrystallization from hexane

**Table 3:** Summary of the reaction sequence for [^18^F]F-FETEM synthesis using GE Tracerlab FXFDG synthesis module.

Synthesis of [^18^F]F-FETEM
1. [^18^F]fluoride trapping on a PS-HCO3- cartridge 2. [^18^F]fluoride desorption by the eluent K2CO3 0.55 mL (6 mg/mL) 3. Addition of catalyst Kryptofix^®^ 222 (15 mg/1 mL acetonitrile) to the reactor vial 4. Azeotropic evaporation 97 °C/1.5 min 5. Addition of precursor (15 mg/1 mL DMSO) to the reactor vial 6. [^18^F]fluorination at 100 °C for 5 min
**Purification of [^18^F]F-FETEM**
7. Transfer and filtration on C18 cartridge with 4 mL WFI:EtOH 7:3 8. [^18^F]F-FETEM desorption by the eluent (4 mL EtOH:WFI 7:3) 9. Collection of [^18^F]F-FETEM in collect vial 10. Final purification with Sep-Pak Plus Alumina N and sterile filtration

## Data Availability

The data presented in this study are available on request from the corresponding author.

## Sample Availability

Samples of the compounds TEM-EtOH, TEM-EtTos and FETEM are not available from the authors.
